# (*E*)-Ethyl 2-cyano-3-(furan-2-yl)acrylate

**DOI:** 10.1107/S1600536812016510

**Published:** 2012-04-21

**Authors:** Rajesh G. Kalkhambkar, D. Gayathri, Vivek K. Gupta, Rajni Kant, Yeon Tae Jeong

**Affiliations:** a Department of Chemistry, Karnatak University’s Karnatak Science College, Dharwad 580 001, Karnataka, India; b Department of Biotechnology, Dr. M.G.R. Educational and Research Institute, Dr. M.G.R. University, Maduravoyal, Chennai 600 095, India; c Post Graduate Department of Physics & Electronics, University of Jammu, Jammu Tawi 180 006, India; d Department of Image Science and Engineering, Pukyong National University, Busan 608 739, Republic of Korea

## Abstract

There are two independent mol­ecules in the asymmetric unit of the title compound, C_10_H_9_NO_3_, in both of which, all non-H atoms except for the methyl C atom lie nearly in the same plane [maximum deviations = 0.094 (3) and 0.043 (2) Å]. In the crystal, each independent mol­ecules is linked by pairs of C—H⋯O inter­actions, generating inversion dimers with *R*_2_^2^(10) ring motifs.

## Related literature

For the synthesis of related compounds, see: Yadav *et al.* (2004 ▶). For related structures, see: Wang & Jian (2008 ▶); Zhang *et al.* (2009 ▶); Ye *et al.* (2009 ▶); Yuvaraj *et al.* (2011 ▶).
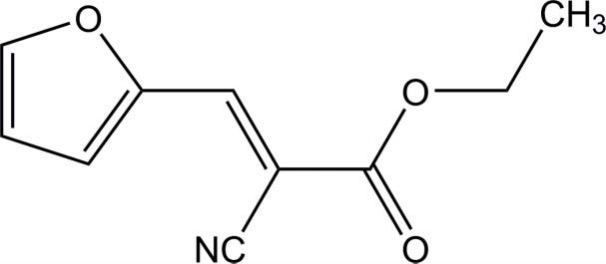


## Experimental

### 

#### Crystal data

C_10_H_9_NO_3_
*M* *_r_* = 191.18
Monoclinic, 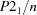

*a* = 4.6611 (2) Å
*b* = 19.8907 (9) Å
*c* = 20.9081 (9) Å
β = 91.988 (4)°
*V* = 1937.28 (15) Å^3^
*Z* = 8
Mo *K*α radiation
μ = 0.10 mm^−1^
*T* = 293 K
0.30 × 0.20 × 0.10 mm

#### Data collection

Oxford Diffraction Xcalibur Sapphire3 diffractometer
Absorption correction: multi-scan (*CrysAlis PRO*; Oxford Diffraction, 2010 ▶) *T*_min_ = 0.933, *T*_max_ = 0.990
12870 measured reflections
4568 independent reflections
2407 reflections with *I* > 2σ(*I*)
*R*_int_ = 0.034

#### Refinement

*R*[*F* ^2^ > 2σ(*F* ^2^)] = 0.066
*wR*(*F* ^2^) = 0.209
*S* = 1.04
4568 reflections
253 parameters
H-atom parameters constrained
Δρ_max_ = 0.26 e Å^−3^
Δρ_min_ = −0.24 e Å^−3^

### 

Data collection: *CrysAlis PRO* (Oxford Diffraction, 2010 ▶); cell refinement: *CrysAlis PRO*; data reduction: *CrysAlis RED* (Oxford Diffraction, 2010 ▶); program(s) used to solve structure: *SHELXS97* (Sheldrick, 2008 ▶); program(s) used to refine structure: *SHELXL97* (Sheldrick, 2008 ▶); molecular graphics: *PLATON* (Spek, 2009 ▶); software used to prepare material for publication: *PLATON*.

## Supplementary Material

Crystal structure: contains datablock(s) I, global. DOI: 10.1107/S1600536812016510/is5119sup1.cif

Structure factors: contains datablock(s) I. DOI: 10.1107/S1600536812016510/is5119Isup2.hkl

Supplementary material file. DOI: 10.1107/S1600536812016510/is5119Isup3.cml

Additional supplementary materials:  crystallographic information; 3D view; checkCIF report

## Figures and Tables

**Table 1:** Hydrogen-bond geometry (Å, °)

*D*—H⋯*A*	*D*—H	H⋯*A*	*D*⋯*A*	*D*—H⋯*A*
C5*A*—H5*A*⋯O2*A*^i^	0.93	2.40	3.242 (3)	151
C5*B*—H5*B*⋯O2*B*^ii^	0.93	2.46	3.320 (3)	153

Symmetry codes: (i) 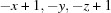; (ii) 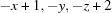.

**Fig. 1.**
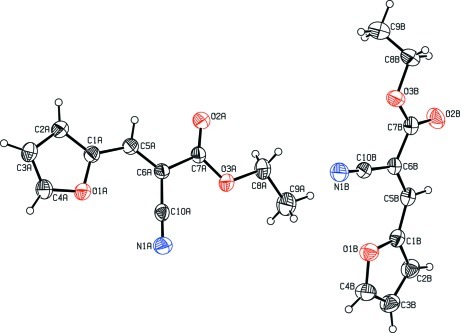
The molecular structure of title compound, showing 30% probability displacement ellipsoids.

**Fig. 2.**
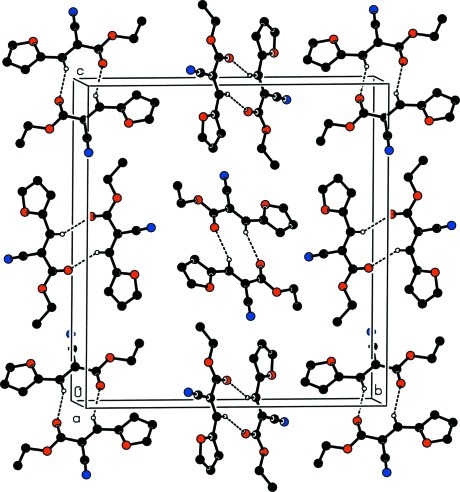
A molecular packing view of the title compound, showing intermolecular interactions. For clarity, hydrogen atoms not involved in the hydrogen bonding have been omitted.

## supplementary crystallographic information

### Comment

Knoevenagel condensation is an important carbon-carbon bond forming reaction in organic synthesis (Yadav *et al.*, 2004). In continuation of our work on nitrogen and oxygen based heterocycles, we herein report the crystal structure of the title compound.

The title compound crystallizes in monoclinic system with two molecules in the asymmetric unit. Bond lengths and bond angles are comparable with the similar crystal structures solved earlier (Zhang *et al.*, 2009; Wang & Jian, 2008; Ye *et al.*, 2009; Yuvaraj *et al.*, 2011). All the non-hydrogen atoms, except the methyl group, lie nearly in the same plane with a maximum out-of-plane deviation of 0.094 (3) and 0.043 (2) Å (r.m.s deviation = 0.04 and 0.024 Å), respectively, for molecules A and B. Difference in the torsion angles C7A—O3A—C8A—C9A [-167.4 (3)°] and C7B—O3B—C8B—C9B [125.3 (4)°] has been observed, indicating the flexibility of the methyl group. The crystal packing is stabilized by C—H···O intermolecular interactions generating the centrosymmetric dimer of *R*_2_^2^(10) ring motif.

### Experimental

A solution of furan-2-aldehyde (1 mol), ethyl cyanoacetate (1.2 mol) and piperidine (0.1 ml) in ethanol (20 ml) was stirred at room temperature for 8 h. After removal of the volatiles *in vacuo*, orange solid was obtained in quantitative yield. A sample for analysis was obtained by recrystallization from EtOAc as pale yellow needles: ^1^H NMR (300 MHz, CDCl_3_) δ p.p.m.: 1.42 (t, 3H, CH_3_), 4.40 (q, 2H, CH_2_), 6.61 (m, 1H, CH), 6.80 (m, 1H, CH), 7.28 (m, 1H, CH), 7.98 (s, 1H, HC=C).

### Refinement

All H-atoms were refined using a riding model [C—H = 0.93 Å and *U*_iso_(H) = 1.2*U*_eq_(C) for aromatic, C—H = 0.97 Å and *U*_iso_ = 1.2*U*_eq_ (C) for CH_2_, and C—H = 0.96 Å and *U*_iso_ = 1.5*U*_eq_(C) for CH_3_].

### Crystal data

**Table:**

C_10_H_9_NO_3_	*F*(000) = 800
*M**_r_* = 191.18	*D*_x_ = 1.311 Mg m^−^^3^
Monoclinic, *P*2_1_/*n*	Mo *K*α radiation, λ = 0.71073 Å
Hall symbol: -P 2yn	Cell parameters from 4295 reflections
*a* = 4.6611 (2) Å	θ = 3.6–29.1°
*b* = 19.8907 (9) Å	µ = 0.10 mm^−^^1^
*c* = 20.9081 (9) Å	*T* = 293 K
β = 91.988 (4)°	Needle, pale yellow
*V* = 1937.28 (15) Å^3^	0.30 × 0.20 × 0.10 mm
*Z* = 8	

### Data collection

**Table:**

Oxford Diffraction Xcalibur Sapphire3 diffractometer	4568 independent reflections
Radiation source: fine-focus sealed tube	2407 reflections with *I* > 2σ(*I*)
Graphite monochromator	*R*_int_ = 0.034
Detector resolution: 16.1049 pixels mm^-1^	θ_max_ = 29.2°, θ_min_ = 3.6°
ω scans	*h* = −6→6
Absorption correction: multi-scan (*CrysAlis PRO*; Oxford Diffraction, 2010)	*k* = −26→24
*T*_min_ = 0.933, *T*_max_ = 0.990	*l* = −27→27
12870 measured reflections	

### Refinement

**Table:**

Refinement on *F*^2^	Primary atom site location: structure-invariant direct methods
Least-squares matrix: full	Secondary atom site location: difference Fourier map
*R*[*F*^2^ > 2σ(*F*^2^)] = 0.066	Hydrogen site location: inferred from neighbouring sites
*wR*(*F*^2^) = 0.209	H-atom parameters constrained
*S* = 1.04	*w* = 1/[σ^2^(*F*_o_^2^) + (0.0908*P*)^2^ + 0.185*P*] where *P* = (*F*_o_^2^ + 2*F*_c_^2^)/3
4568 reflections	(Δ/σ)_max_ < 0.001
253 parameters	Δρ_max_ = 0.26 e Å^−^^3^
0 restraints	Δρ_min_ = −0.24 e Å^−^^3^

### Special details

**Table:**

Geometry. All e.s.d.'s (except the e.s.d. in the dihedral angle between two l.s. planes) are estimated using the full covariance matrix. The cell e.s.d.'s are taken into account individually in the estimation of e.s.d.'s in distances, angles and torsion angles; correlations between e.s.d.'s in cell parameters are only used when they are defined by crystal symmetry. An approximate (isotropic) treatment of cell e.s.d.'s is used for estimating e.s.d.'s involving l.s. planes.
Refinement. Refinement of *F*^2^ against ALL reflections. The weighted *R*-factor *wR* and goodness of fit *S* are based on *F*^2^, conventional *R*-factors *R* are based on *F*, with *F* set to zero for negative *F*^2^. The threshold expression of *F*^2^ > σ(*F*^2^) is used only for calculating *R*-factors(gt) *etc*. and is not relevant to the choice of reflections for refinement. *R*-factors based on *F*^2^ are statistically about twice as large as those based on *F*, and *R*- factors based on ALL data will be even larger.

### Fractional atomic coordinates and isotropic or equivalent isotropic displacement parameters (Å^2^)

**Table:**

	*x*	*y*	*z*	*U*_iso_*/*U*_eq_	
O1A	1.1671 (4)	0.14995 (9)	0.41737 (9)	0.0642 (5)	
O2A	0.5561 (4)	0.03506 (10)	0.58448 (9)	0.0724 (6)	
O3A	0.8013 (4)	0.10818 (9)	0.64695 (8)	0.0617 (5)	
N1A	1.2730 (5)	0.19852 (12)	0.56530 (12)	0.0695 (7)	
C1A	0.9635 (5)	0.10093 (13)	0.41935 (12)	0.0524 (6)	
C2A	0.9058 (6)	0.07717 (15)	0.35931 (13)	0.0658 (8)	
H2A	0.7747	0.0437	0.3478	0.079*	
C3A	1.0809 (7)	0.11270 (15)	0.31803 (14)	0.0709 (8)	
H3A	1.0900	0.1074	0.2740	0.085*	
C4A	1.2328 (7)	0.15591 (16)	0.35497 (14)	0.0747 (9)	
H4A	1.3663	0.1861	0.3397	0.090*	
C5A	0.8457 (5)	0.08207 (13)	0.47874 (12)	0.0520 (6)	
H5A	0.7104	0.0477	0.4761	0.062*	
C6A	0.9001 (5)	0.10636 (12)	0.53795 (11)	0.0468 (6)	
C7A	0.7339 (6)	0.07857 (13)	0.59153 (12)	0.0516 (6)	
C8A	0.6382 (7)	0.08504 (17)	0.70119 (14)	0.0781 (9)	
H8A1	0.4352	0.0835	0.6894	0.094*	
H8A2	0.6995	0.0402	0.7137	0.094*	
C9A	0.6886 (10)	0.13165 (19)	0.75412 (17)	0.1063 (13)	
H9A1	0.5820	0.1173	0.7902	0.159*	
H9A2	0.6274	0.1759	0.7413	0.159*	
H9A3	0.8896	0.1324	0.7658	0.159*	
C10A	1.1070 (6)	0.15760 (13)	0.55250 (12)	0.0511 (6)	
O1B	1.1874 (4)	0.12570 (9)	0.88858 (9)	0.0650 (5)	
O2B	0.5452 (4)	−0.07433 (10)	0.94337 (10)	0.0763 (6)	
O3B	0.7859 (4)	−0.11775 (9)	0.86263 (10)	0.0737 (6)	
N1B	1.2343 (6)	−0.00911 (12)	0.79884 (12)	0.0752 (8)	
C1B	0.9833 (5)	0.11024 (13)	0.93153 (12)	0.0526 (6)	
C2B	0.9368 (6)	0.16376 (14)	0.96945 (14)	0.0654 (8)	
H2B	0.8081	0.1659	1.0024	0.078*	
C3B	1.1182 (7)	0.21538 (15)	0.95001 (15)	0.0717 (8)	
H3B	1.1340	0.2584	0.9672	0.086*	
C4B	1.2645 (7)	0.19017 (15)	0.90153 (17)	0.0732 (8)	
H4B	1.4018	0.2139	0.8795	0.088*	
C5B	0.8538 (5)	0.04531 (13)	0.93108 (12)	0.0545 (7)	
H5B	0.7222	0.0384	0.9629	0.065*	
C6B	0.8908 (5)	−0.00742 (13)	0.89236 (11)	0.0492 (6)	
C7B	0.7208 (6)	−0.06891 (14)	0.90287 (13)	0.0571 (7)	
C8B	0.6333 (9)	−0.18142 (18)	0.86891 (18)	0.0999 (12)	
H8B1	0.7679	−0.2160	0.8829	0.120*	
H8B2	0.4903	−0.1769	0.9013	0.120*	
C9B	0.4999 (11)	−0.2007 (2)	0.8116 (2)	0.1386 (19)	
H9B1	0.4030	−0.2428	0.8173	0.208*	
H9B2	0.6412	−0.2057	0.7796	0.208*	
H9B3	0.3631	−0.1671	0.7981	0.208*	
C10B	1.0847 (6)	−0.00751 (13)	0.84084 (12)	0.0541 (7)	

### Atomic displacement parameters (Å^2^)

**Table:**

	*U*^11^	*U*^22^	*U*^33^	*U*^12^	*U*^13^	*U*^23^
O1A	0.0780 (12)	0.0690 (13)	0.0461 (11)	−0.0191 (10)	0.0102 (9)	−0.0059 (9)
O2A	0.0919 (14)	0.0708 (13)	0.0550 (12)	−0.0275 (11)	0.0091 (10)	0.0000 (10)
O3A	0.0759 (12)	0.0698 (12)	0.0399 (10)	−0.0140 (10)	0.0093 (8)	−0.0011 (8)
N1A	0.0813 (16)	0.0642 (16)	0.0636 (16)	−0.0126 (14)	0.0113 (13)	−0.0078 (12)
C1A	0.0597 (15)	0.0511 (15)	0.0467 (15)	0.0011 (12)	0.0061 (11)	−0.0023 (11)
C2A	0.0812 (19)	0.0655 (18)	0.0510 (16)	−0.0147 (15)	0.0053 (14)	−0.0076 (13)
C3A	0.091 (2)	0.079 (2)	0.0434 (16)	−0.0073 (17)	0.0117 (15)	−0.0046 (14)
C4A	0.095 (2)	0.081 (2)	0.0499 (17)	−0.0172 (17)	0.0225 (16)	−0.0014 (15)
C5A	0.0601 (15)	0.0480 (15)	0.0480 (15)	−0.0011 (12)	0.0049 (11)	−0.0014 (11)
C6A	0.0537 (14)	0.0419 (14)	0.0450 (14)	0.0002 (11)	0.0042 (11)	−0.0007 (10)
C7A	0.0632 (16)	0.0495 (15)	0.0421 (14)	−0.0021 (13)	0.0044 (11)	0.0029 (11)
C8A	0.098 (2)	0.091 (2)	0.0464 (17)	−0.0161 (18)	0.0209 (15)	0.0028 (15)
C9A	0.150 (4)	0.111 (3)	0.060 (2)	−0.021 (3)	0.036 (2)	−0.0106 (19)
C10A	0.0634 (15)	0.0515 (16)	0.0390 (13)	0.0055 (13)	0.0098 (11)	0.0003 (11)
O1B	0.0693 (12)	0.0633 (13)	0.0633 (13)	−0.0045 (10)	0.0132 (9)	−0.0107 (9)
O2B	0.0828 (14)	0.0810 (14)	0.0665 (14)	−0.0095 (11)	0.0257 (11)	0.0105 (11)
O3B	0.1015 (15)	0.0592 (12)	0.0617 (13)	−0.0246 (11)	0.0199 (11)	−0.0061 (10)
N1B	0.0915 (18)	0.0755 (18)	0.0601 (16)	−0.0159 (14)	0.0255 (14)	−0.0079 (13)
C1B	0.0568 (15)	0.0572 (17)	0.0438 (14)	0.0064 (13)	0.0024 (11)	−0.0014 (11)
C2B	0.0736 (18)	0.0630 (19)	0.0596 (18)	0.0083 (15)	0.0041 (14)	−0.0094 (14)
C3B	0.085 (2)	0.0586 (18)	0.071 (2)	0.0061 (16)	−0.0049 (16)	−0.0118 (15)
C4B	0.0759 (19)	0.0613 (19)	0.083 (2)	−0.0098 (16)	0.0064 (16)	−0.0059 (16)
C5B	0.0578 (15)	0.0630 (18)	0.0429 (15)	0.0028 (13)	0.0027 (11)	0.0000 (12)
C6B	0.0553 (14)	0.0531 (15)	0.0392 (13)	−0.0010 (12)	0.0028 (10)	0.0030 (11)
C7B	0.0659 (17)	0.0623 (17)	0.0433 (15)	−0.0005 (14)	0.0016 (12)	0.0049 (13)
C8B	0.151 (3)	0.075 (2)	0.074 (2)	−0.051 (2)	0.011 (2)	0.0059 (18)
C9B	0.220 (5)	0.098 (3)	0.096 (3)	−0.083 (3)	−0.014 (3)	0.009 (2)
C10B	0.0702 (17)	0.0485 (15)	0.0438 (15)	−0.0059 (12)	0.0039 (12)	−0.0017 (11)

### Geometric parameters (Å, º)

**Table:**

O1A—C4A	1.356 (3)	O1B—C4B	1.357 (3)
O1A—C1A	1.362 (3)	O1B—C1B	1.366 (3)
O2A—C7A	1.204 (3)	O2B—C7B	1.203 (3)
O3A—C7A	1.328 (3)	O3B—C7B	1.327 (3)
O3A—C8A	1.461 (3)	O3B—C8B	1.461 (4)
N1A—C10A	1.148 (3)	N1B—C10B	1.140 (3)
C1A—C2A	1.360 (3)	C1B—C2B	1.349 (4)
C1A—C5A	1.425 (3)	C1B—C5B	1.425 (4)
C2A—C3A	1.400 (4)	C2B—C3B	1.399 (4)
C2A—H2A	0.9300	C2B—H2B	0.9300
C3A—C4A	1.341 (4)	C3B—C4B	1.338 (4)
C3A—H3A	0.9300	C3B—H3B	0.9300
C4A—H4A	0.9300	C4B—H4B	0.9300
C5A—C6A	1.345 (3)	C5B—C6B	1.340 (3)
C5A—H5A	0.9300	C5B—H5B	0.9300
C6A—C10A	1.429 (4)	C6B—C10B	1.430 (3)
C6A—C7A	1.490 (3)	C6B—C7B	1.478 (4)
C8A—C9A	1.457 (4)	C8B—C9B	1.385 (5)
C8A—H8A1	0.9700	C8B—H8B1	0.9700
C8A—H8A2	0.9700	C8B—H8B2	0.9700
C9A—H9A1	0.9600	C9B—H9B1	0.9600
C9A—H9A2	0.9600	C9B—H9B2	0.9600
C9A—H9A3	0.9600	C9B—H9B3	0.9600
			
C4A—O1A—C1A	105.8 (2)	C4B—O1B—C1B	105.5 (2)
C7A—O3A—C8A	115.1 (2)	C7B—O3B—C8B	117.1 (2)
C2A—C1A—O1A	109.7 (2)	C2B—C1B—O1B	109.8 (2)
C2A—C1A—C5A	130.0 (3)	C2B—C1B—C5B	130.0 (3)
O1A—C1A—C5A	120.3 (2)	O1B—C1B—C5B	120.3 (2)
C1A—C2A—C3A	107.0 (3)	C1B—C2B—C3B	107.3 (3)
C1A—C2A—H2A	126.5	C1B—C2B—H2B	126.4
C3A—C2A—H2A	126.5	C3B—C2B—H2B	126.4
C4A—C3A—C2A	106.0 (3)	C4B—C3B—C2B	105.9 (3)
C4A—C3A—H3A	127.0	C4B—C3B—H3B	127.1
C2A—C3A—H3A	127.0	C2B—C3B—H3B	127.1
C3A—C4A—O1A	111.5 (3)	C3B—C4B—O1B	111.5 (3)
C3A—C4A—H4A	124.3	C3B—C4B—H4B	124.2
O1A—C4A—H4A	124.3	O1B—C4B—H4B	124.2
C6A—C5A—C1A	129.9 (2)	C6B—C5B—C1B	130.5 (2)
C6A—C5A—H5A	115.1	C6B—C5B—H5B	114.7
C1A—C5A—H5A	115.1	C1B—C5B—H5B	114.7
C5A—C6A—C10A	123.8 (2)	C5B—C6B—C10B	123.7 (2)
C5A—C6A—C7A	118.2 (2)	C5B—C6B—C7B	118.5 (2)
C10A—C6A—C7A	118.0 (2)	C10B—C6B—C7B	117.9 (2)
O2A—C7A—O3A	124.5 (2)	O2B—C7B—O3B	123.8 (3)
O2A—C7A—C6A	123.2 (2)	O2B—C7B—C6B	124.1 (3)
O3A—C7A—C6A	112.2 (2)	O3B—C7B—C6B	112.1 (2)
C9A—C8A—O3A	108.3 (3)	C9B—C8B—O3B	111.6 (3)
C9A—C8A—H8A1	110.0	C9B—C8B—H8B1	109.3
O3A—C8A—H8A1	110.0	O3B—C8B—H8B1	109.3
C9A—C8A—H8A2	110.0	C9B—C8B—H8B2	109.3
O3A—C8A—H8A2	110.0	O3B—C8B—H8B2	109.3
H8A1—C8A—H8A2	108.4	H8B1—C8B—H8B2	108.0
C8A—C9A—H9A1	109.5	C8B—C9B—H9B1	109.5
C8A—C9A—H9A2	109.5	C8B—C9B—H9B2	109.5
H9A1—C9A—H9A2	109.5	H9B1—C9B—H9B2	109.5
C8A—C9A—H9A3	109.5	C8B—C9B—H9B3	109.5
H9A1—C9A—H9A3	109.5	H9B1—C9B—H9B3	109.5
H9A2—C9A—H9A3	109.5	H9B2—C9B—H9B3	109.5
N1A—C10A—C6A	178.8 (3)	N1B—C10B—C6B	177.9 (3)
			
C4A—O1A—C1A—C2A	0.1 (3)	C4B—O1B—C1B—C2B	0.1 (3)
C4A—O1A—C1A—C5A	−179.9 (2)	C4B—O1B—C1B—C5B	179.8 (2)
O1A—C1A—C2A—C3A	0.1 (3)	O1B—C1B—C2B—C3B	0.0 (3)
C5A—C1A—C2A—C3A	−179.9 (3)	C5B—C1B—C2B—C3B	−179.7 (3)
C1A—C2A—C3A—C4A	−0.3 (3)	C1B—C2B—C3B—C4B	−0.1 (3)
C2A—C3A—C4A—O1A	0.4 (4)	C2B—C3B—C4B—O1B	0.2 (4)
C1A—O1A—C4A—C3A	−0.3 (4)	C1B—O1B—C4B—C3B	−0.2 (3)
C2A—C1A—C5A—C6A	−178.9 (3)	C2B—C1B—C5B—C6B	177.4 (3)
O1A—C1A—C5A—C6A	1.1 (4)	O1B—C1B—C5B—C6B	−2.2 (4)
C1A—C5A—C6A—C10A	−2.1 (4)	C1B—C5B—C6B—C10B	0.5 (4)
C1A—C5A—C6A—C7A	177.5 (2)	C1B—C5B—C6B—C7B	−179.0 (2)
C8A—O3A—C7A—O2A	−1.0 (4)	C8B—O3B—C7B—O2B	−0.2 (4)
C8A—O3A—C7A—C6A	177.8 (2)	C8B—O3B—C7B—C6B	179.4 (3)
C5A—C6A—C7A—O2A	0.8 (4)	C5B—C6B—C7B—O2B	1.7 (4)
C10A—C6A—C7A—O2A	−179.5 (2)	C10B—C6B—C7B—O2B	−177.8 (3)
C5A—C6A—C7A—O3A	−178.0 (2)	C5B—C6B—C7B—O3B	−177.9 (2)
C10A—C6A—C7A—O3A	1.6 (3)	C10B—C6B—C7B—O3B	2.6 (3)
C7A—O3A—C8A—C9A	−167.4 (3)	C7B—O3B—C8B—C9B	125.3 (4)

### Hydrogen-bond geometry (Å, º)

**Table:**

*D*—H···*A*	*D*—H	H···*A*	*D*···*A*	*D*—H···*A*
C5*A*—H5*A*···O2*A*^i^	0.93	2.40	3.242 (3)	151
C5*B*—H5*B*···O2*B*^ii^	0.93	2.46	3.320 (3)	153

Symmetry codes: (i) −*x*+1, −*y*, −*z*+1; (ii) −*x*+1, −*y*, −*z*+2.

## References

[bb1] Oxford Diffraction (2010). *CrysAlis PRO* and *CrysAlis RED* Oxford Diffraction Ltd, Yarnton, England.

[bb2] Sheldrick, G. M. (2008). *Acta Cryst.* A**64**, 112–122. 10.1107/S0108767307043930 18156677

[bb3] Spek, A. L. (2009). *Acta Cryst.* D**65**, 148–155. 10.1107/S090744490804362X PMC2631630 19171970

[bb4] Wang, J.-G. & Jian, F.-F. (2008). *Acta Cryst.* E**64**, o2145. 10.1107/S1600536808032984 PMC2959598 21581005

[bb5] Yadav, J. S., Subba Reddy, B. V., Basak, A. K., Visali, B., Narsaiah, A. V. & Nagaiah, K. (2004). *Eur. J. Org. Chem.* pp. 546–551.

[bb6] Ye, Y., Shen, W.-L. & Wei, X.-W. (2009). *Acta Cryst.* E**65**, o2636. 10.1107/S1600536809039518 PMC2971297 21578251

[bb7] Yuvaraj, H., Gayathri, D., Kalkhambkar, R. G., Gupta, V. K. & Rajnikant, (2011). *Acta Cryst.* E**67**, o2135. 10.1107/S1600536811028790 PMC3213575 22091152

[bb8] Zhang, S.-J., Zheng, X.-M. & Hu, W.-X. (2009). *Acta Cryst.* E**65**, o2351. 10.1107/S1600536809035132 PMC2970386 21577820

